# Disability Studies and Disability Evaluation in Healthcare—Themes and Challenges Moving Forward

**DOI:** 10.3390/healthcare14081016

**Published:** 2026-04-13

**Authors:** Debra A. Harley, Si-Yi Chao

**Affiliations:** Department of Early Childhood, Special Education, and Counselor Education, University of Kentucky, 229 Taylor Education Building, 597 S Upper St, Lexington, KY 40506-0001, USA; siyichao7185@uky.edu

## 1. Introduction

Persons with disabilities experience significant barriers to accessing healthcare, yet the gap between disability studies and healthcare service delivery remains a persistent structural problem. The number of individuals living with disabilities is growing, which is attributed to factors such as population increase, aging, and medical advances that increase survival rates and prolong life; this has increased the demand for health services [1]. However, demand alone does not translate into access. Persons with disabilities and their families experience significant barriers to accessing inclusive healthcare on a global scale [2]. Building health systems that genuinely respond to the complexity of disability-related needs requires a deliberate, coordinated effort across research, policy, and practice [3].

Among the most immediate human consequences of inadequate systemic support is the burden carried by family caregivers. Caregivers of persons with disabilities, especially those supporting children with intellectual and developmental disabilities (IDD), bear substantial burden and stress in managing the demands of daily care [4]. Having a child with special health and care needs creates multiple challenges for families as a unit, for this individual child, and for the parents and/or caregivers. These children require levels of medical care and daily assistance that exceed those of children without disabilities, placing demands on families that adversely affect their work–life balance [5]. Social support is related to better long-term care planning for a child or adult with IDD, but fewer than 40% of caregivers report having the social support they need [6].

The challenges do not diminish as individuals with disabilities age and, in many respects, they intensify with time. People with IDD are living longer and experiencing life transitions, such as retirement, that the existing service and policy frameworks were not designed to address [7]. Functional aging among people with IDD accelerates relative to the general population, and the mismatch between chronological age and functional capacity exposes a structural inadequacy in how care systems categorize and respond to needs. Although the importance of interdisciplinary collaboration in developing planning, pathways, and the integration of disability within the broader healthcare services is recognized, the extent to which disability studies and disability evaluation research examine disability inclusion in healthcare and from an interdisciplinary perspective is limited. Building on efforts to create a more inclusive healthcare and recognizing that a variety of people with chronic illnesses and disabilities exist along a spectrum of health and function, Gulley et al. [8] argue that the existing frameworks for addressing the health of persons with chronic conditions and disabilities require fundamental evaluation and reform, particularly for those whose care needs are ongoing or elevated. This Special Issue enters this broader discourse precisely when the consequences of inadequate response—for caregivers, aging adults with disabilities, and marginalized communities worldwide—remain profound and pressing.

## 2. An Overview of Published Articles in This Special Issue

The aim of this Special Issue, “Disability Studies and Disability Evaluation”, is to evaluate how disability studies impacts service delivery and the health and social outcomes of people with disability through an examination of disability awareness, perceptions of healthcare providers, and the influence of social factors on quality of life. Emphasis is placed on the implications of individual, psychosocial, and organizational elements of improving health and mental health outcomes among people with chronic illness and disability. The articles included in this Special Issue address a broad spectrum of topics within this framework and reflect perspectives including caregiver burden in families; interdisciplinary research between critical disability studies and healthcare; person-centered management in disability services; age disparities between disability types; intersecting identities in college students across ethnicities; enhancing social, financial and health service inclusivity; the support of workers with intellectual and developmental disabilities transitioning into retirement; disability-inclusive approaches within neglected tropical disease programming; and the structural evolution of inclusive healthcare systems for children with disabilities. The nine articles trace a path from family caregiving and research epistemology through systemic inequities in service delivery, workforce participation, and global health infrastructure.

Caregivers of children with disabilities carry a disproportionate burden, with diminished support and reduced coping capacity. Garcia-Grau et al. [Contributor 1] present a fuzzy-set qualitative comparative analysis of causal configurations of psychosocial conditions associated with both high and low levels of caregiver burden in families of children receiving early intervention services. Low family confidence emerges as the central driver of elevated burden, while parental self-efficacy and social support retain protective value regardless of a child’s functional level [Contributor 1]. Toman et al. [Contributor 2] raise critical questions for researchers when conducting disability research and challenges the oppositional training of professionals in disability studies and the healthcare and medical research fields, highlighting that continued non-collaboration inflicts measurable harm on the populations that both fields claim to serve and that repositioning disabled individuals as active research partners is an ethical requirement, rather than a programmatic aspiration. Disability services are recognized as being person-centered approaches to promote positive outcomes for individuals with disabilities, of which adequate allocation of resources is required to meet the planned services [Contributor 2]. Bianchi et al. [Contributor 3] investigate how individual and contextual factors shape resource use in individualized support planning of those with intellectual and developmental disabilities (IDD) who receive services in residential, family, or independent living. As individuals with developmental disabilities grow older, the effects of their functional age are accelerated compared to their non-IDD counterparts [Contributor 3]. Jeong [Contributor 4] measures the disparity in functional ability and chronic illness prevalence between adults with developmental disabilities and other disabilities, finding activity limitations that current policy frameworks are inadequate to respond to and for which function-based care models are urgently needed [Contributor 4].

Structural disadvantage extends beyond care and service systems. Students with disabilities are underrepresented in college and face compounded disadvantages in career-related factors. Chao and Wilson [Contributor 5] explore the influence of intersecting disabilities and ethnic identities on career decision self-efficacy, career outcome expectations, perceived career barriers, and support among college students with disabilities from diverse racial and ethnic backgrounds, indicating that students from underrepresented ethnic groups face markedly greater career-related barriers than their European American peers and that culturally responsive interventions affirming multiple identities are structurally necessary [Contributor 5]. Access to social support and financial means can influence the inclusion of persons with disabilities in service utilization. Alturif et al. [Contributor 6] explore caregivers’ perspectives on awareness, perceived barriers, and accessibility of social and financial services for people with disabilities in Saudi Arabia, documenting that over 70% of caregivers remain unaware of available supports, a gap deepened by technological barriers, administrative complexity, and the near-absence of decentralized outreach in rural areas [Contributor 6]. The effects of disability across the lifespan elicit different adjustments. Sanchez et al. [Contributor 7] focus on needs of aging workers with IDD who experience a declining work ability because of aging and disability and who transition to retirement, underscoring that flexible workplace accommodations and individualized planning across families, employers, and professionals are critical for dignified aging [Contributor 7]. The most marginalized populations are disproportionately faced with disease and illness that are further perpetuated by structural, social, and political attributes of exclusion. Juma et al. [Contributor 8] examine the concept of inclusion within neglected tropical disease (NTD) programming, with a particular focus on intersecting forms of marginalization, including poverty, gender, disability, and displacement. The authors contend that disability-inclusive strategies and community empowerment must be embedded throughout health systems, rather than treated as peripheral concerns [Contributor 8]. Children with disabilities continue to face systemic challenges in healthcare. Gulgosteren et al. [Contributor 9] employ bibliometric methods and analyze the structure and evolution of the scientific literature on inclusive healthcare systems for children with disabilities, identifying growing academic attention to neurodevelopmental disorders and family-centered care since 2015. Still, the dominance of high-income countries in the literature remains a structural barrier to equitable, community-based care globally [Contributor 9].

## 3. Conclusions

Collectively, the contributors in this Special Issue call attention to the current state of evaluation or measurement of disabilities, as well as a need for coordinated interdisciplinary research between disability studies and healthcare. Several themes emerged from the contribution in this Special Issue:

○ The healthcare needs of children and adults with intellectual and developmental disabilities;
○ Support for caregivers of persons with disabilities;
○ The intersectionality of disability and demographic characteristics;
○ The social determinants of inclusion of persons with disabilities in healthcare service utilization;
○ Life transitions of people with disabilities.

Disability is extraordinarily diverse, yet across different national contexts and population groups, people with disabilities experience remarkably consistent barriers when seeking healthcare, including exclusion, lack of autonomy in decision-making about their health, healthcare costs, poor availability of services, and physical and systematic inaccessibility [3]. The gap in disability studies and evaluation in healthcare causes consideration for a comprehensive, inclusive approach that accounts for people with disabilities as active participants, integrates family members and informal supports, aligns planned services with real-world needs, and attends to the full range of transitions across the lifespan. Movement from policy to practice is a foundational requirement of any comprehensive disability healthcare approach. The present Special Issue addresses this gap through building determinants of enablement that can lead to better management of healthcare services for people with disabilities and their families.

Moving forward, to better address the interplay between disability and healthcare, future research should be collaborative across disability studies and healthcare disciplines, and structurally integrated in design, methodology, and dissemination, rather than parallel or nominally interdisciplinary. A lifespan perspective is essential because the sequence and progression of many types of disabilities are expected to continue indefinitely, and frameworks that only address discrete life stages will remain inadequate to the realities that people with disabilities actually face. Persons with disabilities continue to encounter considerable barriers when accessing healthcare services, which negatively affects their chances of achieving their highest attainable standard of living and might contribute to their shorter life expectancy and poorer health outcomes than people without disabilities [8]. Sustained commitment to collaborative, lifespan-oriented, and structurally integrated research remains the most defensible means of achieving the equitable healthcare outcomes that people with disabilities deserve.
